# Association Between Polymorphism (5-HTTLPR) of the Serotonin Transporter Gene and Behavioral Response to Unfair Distribution

**DOI:** 10.3389/fnbeh.2022.762092

**Published:** 2022-03-16

**Authors:** Kuniyuki Nishina, Qiulu Shou, Hidehiko Takahashi, Masamichi Sakagami, Miho Inoue-Murayama, Haruto Takagishi

**Affiliations:** ^1^Graduate School of Human Sciences, Osaka University, Osaka, Japan; ^2^Graduate School of Brain Sciences, Tamagawa University, Tokyo, Japan; ^3^Graduate School of Medical and Dental Sciences, Medical and Dental University, Tokyo, Japan; ^4^Brain Science Institute, Tamagawa University, Tokyo, Japan; ^5^Wildlife Research Center, Kyoto University, Kyoto, Japan

**Keywords:** economic game, serotonin transporter gene, 5-HTTLPR, unfairness, third-party punishment

## Abstract

Behavioral responses to unfair distribution have been measured mainly using the Ultimatum Game (UG). Recent studies examining the biological basis of behavioral responses to unfair distribution have focused attention on the role of the serotonin transporter gene. However, studies, to date, have been conducted on non-Asians, and it has not been confirmed whether similar results can be seen in other ethnic groups. It has also been shown that behavioral responses to unfair distribution are not only seen in the case of victims themselves but also in the case of third parties not directly affected. This study aimed to determine whether the results of the previous study would be replicated in an Asian population and whether the serotonin transporter gene would also be associated with behavior toward unfair distribution by third parties. We examined the association between polymorphisms (5-HTTLPR) of the serotonin transporter gene and participants’ behavior in the UG and the third-party punishment game (TPPG). The results did not show an association between punishment for unfair proposals in the TPPG and genetic polymorphisms, while participants with the SL/LL genotype were more likely to reject unfair offers in the UG than those with the SS genotype. These results indicate that 5-HTTLPR is associated with behavior when unfair intentions are directed at oneself.

## Introduction

Punishing those who violate social norms such as unfair distribution of resources plays an important role in maintaining a cooperative society (Yamagishi, 1986; Fehr and Gächter, 2002; Fehr and Fischbacher, 2003; Bowles and Gintis, 2013). Behavioral responses to unfair distribution have been examined using a two-person economic game, called the Ultimatum Game (UG; Güth et al., 1982). One player (the proposer) receives some money from the experimenter and suggests how to divide the money between themself and the other player (the responder). After the proposer has made a decision, the responder decides whether to accept or reject the offer. If the responder accepts the offer, both players receive the offered amount. If the responder rejects the offer, neither player receives anything. In previous research, offers of 30% or less to responders are often rejected (Camerer, 2003), and rejection of unfair offers are presumably based on aversion to inequality (Fehr and Gintis, 2007).

In recent years, attention has been focused on the biological basis of behavioral responses to unfair distribution, and the neurotransmitter serotonin (5-HT) has been shown to play an important role in rejecting unfair offers in the UG (Crockett et al., 2008, 2010a; Emanuele et al., 2008). 5-HT is a monoamine neurotransmitter that controls impulsivity and the regulation of emotions such as aggression, provocation, and anger (Krakowski, 2003). Many studies have shown that low 5-HT levels increase the rejection of unfair offers. For instance, Crockett et al. (2008) found that temporarily lowering 5-HT via tryptophan depletion increases the rejection of unfair offers in the UG. Further, studies have shown that an increase in rejection is positively related to an increase in making impulsive choices due to a temporary lowering of the 5-HT (Crockett et al., 2010b), implying that rejection of unfair offers in the UG is associated with impulsivity. In line with these studies, researchers have found that individuals with lower levels of serotonergic activity measured by platelet 5-HT concentrations tend to reject unfair offers in the UG (Emanuele et al., 2008) and that individuals with enhanced 5-HT via citalopram (a selective 5-HT reuptake inhibitor) are less likely to reject unfair offers in the UG (Crockett et al., 2010a).

The relationship between the serotonin transporter (5-HTT), which is involved in serotonin functioning, and rejection of unfair offers has also been investigated. Takahashi et al. (2012) assessed 5-HTT levels in the brain using positron emission tomography scans and showed that people with a low level of 5-HTT in the dorsal raphe nucleus tend to reject unfair offers in the UG. In addition, a polymorphism (5-HTTLPR) in the promoter region of the serotonin transporter gene (*SLC6A4*) on chromosome 17 (17q.11.2) has been associated with rejection in the UG. Polymorphisms (5-HTTLPR) in *SLC6A4* are classified as LL with two long repeat alleles, SS with two short repeat alleles, and SL with each of the two. It has been shown that the L-allele has a higher expression of the 5-HTT and a higher rate of 5-HT uptake than the S-allele (Heils et al., 1996; Lesch et al., 1996; Greenberg et al., 1999). These results suggest that 5-HT levels are higher in L-allele carriers than in S-allele carriers. Two studies have shown that individuals with the LL genotype in 5-HTTLPR reject unfair UG offers more than those with the S-allele (Enge et al., 2017; Gärtner et al., 2018).

An important issue in the study of 5-HTTLPR is the cultural differences in the genotype distribution. Previous cross-cultural studies have shown that LL genotype is more common in people of European origin and less common in Asians. Conversely, the SS genotype is less common in people of European origin and more common in Asians (Gelernter et al., 1997; Chiao and Blizinsky, 2010; Ishii et al., 2014). Previous studies examining the association between 5-HTTLPR and rejection of unfair UG offers have been conducted in non-Asians; these studies have compared LL and SL/SS genotypes to show an association. It will be important to understand the role of 5-HTTLPR in rejecting unfair offers to see if the same results can be replicated by comparing SS and SL/LL genotypes.

In addition, behavioral responses to inequitable distribution are not limited to direct victims of unfairness but are also found in third-party situations. Previous research has shown that third parties who are not directly affected by unfair distribution tend to pay their own money to reduce the money of those who have made unfair distributions (Fehr and Fischbacher, 2004). As in this study, the behavioral response to unfair distribution may be in the position of a direct victim or a third party, but the association with 5-HTTLPR has been examined only in the former, not the latter.

In order to accurately examine the role of the serotonin transporter in behavioral responses to unfair distribution, it is necessary to examine and compare the association between the two different positions and the 5-HTTLPR. The primary purpose of this study is to see if the results of previous studies can be replicated in Asian participants. The second purpose is to examine whether behavioral responses to unfair distribution as a third party as well as being a direct victim of unfair distribution are associated with 5-HTTLPR.

## Materials and Methods

### Experimental Setting

The experiments reported in this manuscript were conducted as part of a project aimed at elucidating the psychological and neural basis of human prosocial behavior (details can be found at http://www.human-sociality.net/english/). At the beginning of the project, we created a pool of 500 participants (including both men and women) between 20 and 59 years old, living in the Tokyo area. We conducted 10 experiments with the participants from 2012 to 2020. In the experiments, we collected results from economic games, cognitive tasks, questionnaires, MRI data, hormone assays, and genetic data. The results of this project have already been summarized in several manuscripts, but this is the first report on serotonin transporter gene polymorphisms and behavioral responses to unfair offers. In each experiment, 500 participants were recruited, but the number of participants in each experiment varied depending on the participants’ personal circumstances. The number of participants for each experiment is provided in the sections describing each experiment type.

### Ultimatum Game

The UG experiment took place between December 2013 and February 2014, with a total of 441 adults (242 women) taking part. The UG is an economic game played in pairs. One player (the proposer) receives money (JPY 1,500) from the experimenter and suggests to the other player (the responder) a plan to divide that money between the two in 10% increments. The responder then decides whether to accept or reject the offer. If the responder accepts the offer, both players receive the money offered by the proposer. If the responder rejects the offer, neither player receives anything. The responder’s behavior is measured using the strategy method, in which the responder decides whether to accept or reject all offers from the proposer. In the experiment, all participants first decided to be proposers, and then responders with different partners. Based on the results of previous studies showing that proposals for 30% and less than 30% of the endowment are often rejected (Camerer, 2003; Takahashi et al., 2012), we defined the same criteria as an unfair offer in this study.

### Third-Party Punishment Game

Four hundred and seventy adults (243 women) played the TPPG between May 2014 and July 2014. The TPPG is an economic game played by three players. First, the proposer decided how to divide the money (JPY 1,500) received from the experimenter between themself and the recipient in 10% increments. The recipients received the money allotted to them by the proposer. The third party then decided how much of their own money they would spend to punish the proposer (within JPY 0–500, in increments of 10) for the perceived unfairness of their choice. Three times the amount paid by the third party for punishment was deducted from the proposer. In the experiment, all participants performed the task first as proposers, then as recipients, and finally as the third party, switching partners each time. Similar to the UG, we defined proposals made by proposers to recipients of less than 30% as unfair offers.

### Genotyping

The buccal cells of 441 adults (223 women) were collected between October 2014 and January 2015. DNA was extracted from the buccal mucosa cells using the DNeasy Blood and Tissue Kit (QIAGEN, Tokyo, Japan). DNA was amplified by PCR using the following primers (5′-GGCGTTGCCGCTCTGAATGC-3′ and 5′-GAGGGACTGAGCTGGACAACCAC-3′), and the PCR products were subjected to electrophoresis to determine the genotype of 5-HTTLPR. Further, PCR amplification was performed under the same conditions as those used in previous studies (e.g., Inoue-Murayama et al., 2000). We classified 14 repeats as short (S) allele and 15 or more repeats as long (L) allele based on the length of the number of repeats in the 5-HTTLPR.

### Statistical Analysis

The analyses were conducted using SAS 9.4, and RStudio version 1.4. As the distributions of the average rejection rate of unfair offers in the UG and the average amount of punishment in the TPPG were not normally distributed (*p*s < 0.0001), we used logistic regression analysis with a dummy variable of whether they rejected once or punished once as the objective variable. Robust standard errors were used for standard errors. Age and sex were added to the explanatory variables as control variables because they are confounders of behavioral responses to unfair distribution. Since this study dealt with two types of punishment, UG and TPPG, the significance level α for analyzing each type of punishment was set at 0.025. In the experiment, the same participants participated in UG and TPPG play and buccal mucosa cell collection on different days. However, due to the participants’ circumstances, the number of participants differed each day. Finally, 421 adults (212 females, mean age 41.2 years, SD = 10.3) with complete UG, TPPG, and genetic data were analyzed. All participants were Asians with Japanese as their native language.

## Results

### Genotype Distribution

The distribution of genotypes in the 5-HTTLPR was as follows: 242 (57.5%) had two short alleles (SS), 156 (37.1%) had one long allele and one short allele (SL), and 23 (5.4%) had two long alleles (LL). The distribution of this genotype did not differ significantly from the Hardy-Weinberg equilibrium [χ^2^(1) = 0.108, *p* = 0.742]. As the number of participants with the LL genotype was extremely small, the SL and LL genotypes were grouped together for comparison with the SS genotype. We compared the mean values of punishment in the SL and LL groups and found no significant difference between their game performance [UG: SL genotype, *M* = 0.58, SD = 0.45; LL genotype, *M* = 0.42, SD = 0.50; *t*(177) = 1.56; *p* = 0.121, TPPG: SL genotype, *M* = 50.8, SD = 81.5; LL genotype, *M* = 55.4, SD = 81.9; *t*(177) = 0.25; *p* = 0.801].

### Ultimatum Game

**FIGURE 1:**
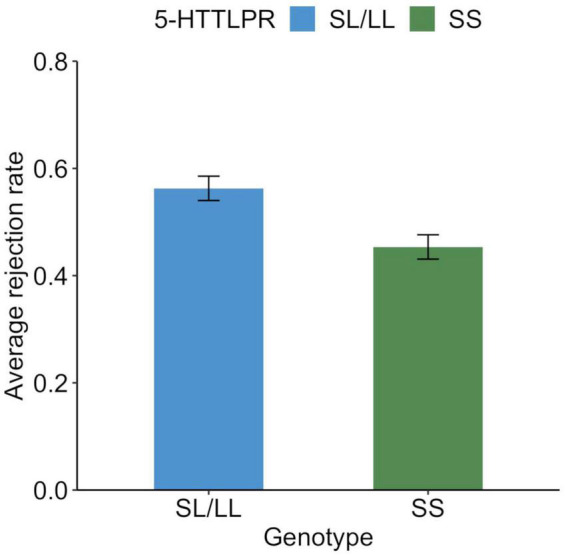
Behavioral response to unfair offers in the ultimatum game among each genotype. Average rejection rate of unfair proposals (30% and less than 30% to responders) in the ultimatum game. Error bars indicate the standard error.

**FIGURE 2:**
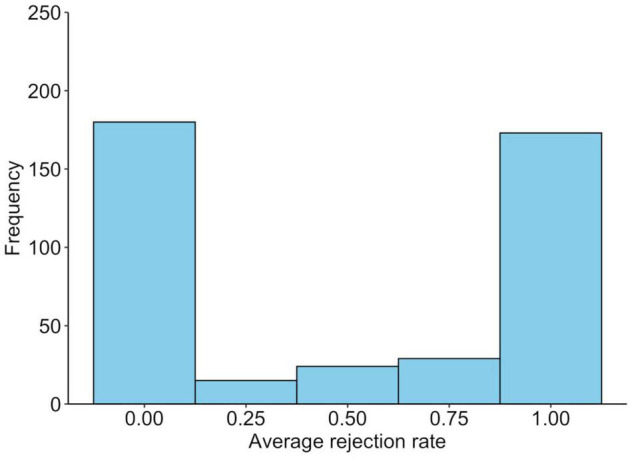
Distribution of the behavioral responses to unfair offers in the ultimatum game. The average rejection rate of unfair offers in ultimatum games.

The average rejection rate for unfair offers was 0.50 (SD = 0.47), while 241 participants (57.2%) rejected at least one offer. Those who rejected the unfair offer at least once were older than those who did not reject it at all [*t*(419) = 2.2, *p* = 0.032], but this was not related to sex [χ^2^(1) = 0.5, *p* = 0.473]. The average rejection rates of unfair offers for the SS and SL/LL genotypes are shown in Figure 1, whereas the distribution of the average rejection rates is shown in Figure 2. A logistic regression analysis with the variable of whether the unfair offers were rejected at least once (rejected = 1) as the objective variable, and age, gender (men = 1), and SS genotype (SS = 1) as explanatory variables found a significant effect of SS genotype (*b* = −0.23, SE = 0.10, Wald χ^2^ = 5.1, *p* = 0.024, odds ratio = 0.63, 95% CI [0.43, 0.94]). The effects of age (*b* = −0.02, SE = 0.01, Wald χ^2^ = 4.6, *p* = 0.035, odds ratio = 0.98, 95% CI [0.96, 0.99]) and gender were not significant (*b* = −0.08, SE = 0.10, Wald χ^2^ = 0.7, *p* = 0.042, odds ratio = 0.85, 95% CI [0.58, 1.26]).

### Third-Party Punishment Game

**FIGURE 3:**
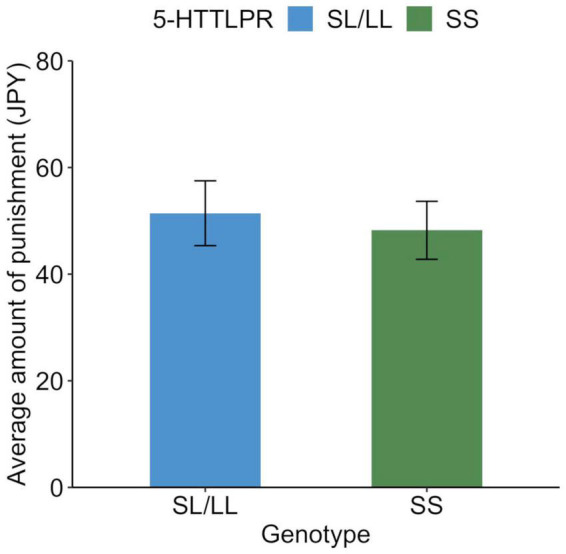
Behavioral response to unfair distribution in the third-party punishment game among each genotype. Average amount of punishment for unfair distribution (30% and less than 30% to recipient) in the third-party punishment game. Error bars indicate the standard error.

**FIGURE 4:**
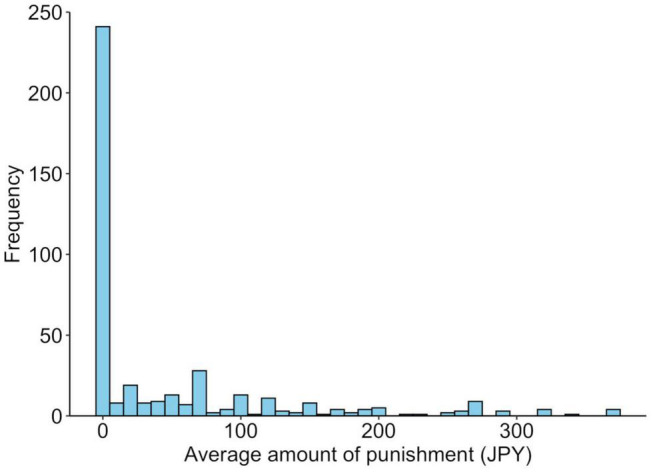
Distribution of the behavioral responses to unfair distribution in the third-party punishment game. The average punishment amount for unfair distribution in the third-party punishment game.

The average amount of punishment was JPY 49.6 (SD = 83.1). Of all the participants, 188 (44.7%) spent at least JPY 1 at least once for punishment. The remaining 233 people (55.3%) did not use money for punishment. Those who were punished were older than those who were not punished [*t*(419) = 3.5, *p* = 0.0005], but there was no association with gender [χ^2^(1) = 1.1, *p* = 0.296]. The average punishments for each genotype are shown in Figure 3, and their distributions are shown in Figure 4. Similar to the analysis of the UG, we conducted a logistic regression analysis in which the objective variable was whether or not proposers received punishment (punished = 1). The results showed that although the effect of age (*b* = −0.03, SE = 0.01, Wald χ^2^ = 11.8, *p* = 0.0006, odds ratio = 0.97, 95% CI [0.95, 0.99]) was significant, the effects of the SS genotype (*b* = −0.11, SE = 0.10, Wald χ^2^ = 1.3, *p* = 0.256, odds ratio = 0.80, 95% CI [0.54, 1.18]) and gender (*b* = −0.12, SE = 0.10, Wald χ^2^ = 1.4, *p* = 0.243, odds ratio = 0.79, 95% CI [0.54, 1.17]) were not significant.

## Discussion

This study examined how polymorphisms in the serotonin transporter gene (5-HTTLPR) are associated with behavioral responses to unfair offers in economic games in Asian populations. The results showed that people with the SS genotype were less likely to reject unfair offers in the UG than those with the SL/LL genotype. The study results are similar to those of previous studies in non-Asians (Enge et al., 2017; Gärtner et al., 2018), even when the genotype classification is used for Asians (comparing LL and SL together with SS). In previous studies (Enge et al., 2017; Gärtner et al., 2018), people with the LL genotype were more likely to reject unfair proposals in the UG than those with the SL/SS genotype (LL > SL/SS). In this study, people with the LL/SL genotype were more likely to reject unfair proposals in the UG than those with the SS genotype (LL/SL > SS). The results of these studies show that rejection changes in a sequential manner, with the SS type having the lowest rejection rate, followed by the SL type, and the LL type having the highest rejection rate. For such genes with cultural differences in the distribution of genotypes, studies in various regions will be important because the classification may differ depending on the target sample.

In the TPPG, there was no association between punishment for unfair offers and the genetic polymorphism of 5-HTTLPR. These results have important implications for examining the role of serotonin in behavioral responses to unfair offers. What factors specific to the UG are not present in the TPPG? Previous studies have shown that the primary motivation for behavioral responses to unfair offers in the UG is not the enforcement of social norms but rather a decrease in the status of the target proposer relative to one’s own status (Burnham, 2007; Yamagishi et al., 2009, 2012). Unfair proposals in the UG can be interpreted as an act of the proposer demonstrating their superiority to the participants. The rejection of unfair offers may reflect spiteful behavior when confronted with displays of dominance. Such a motivation is clearly different from the altruistic motivation of reinforcing social norms; indeed, it has been shown that rejection of unfair offers in the UG is not associated with altruistic or fair behavior (Yamagishi et al., 2012). The behavioral response to unfair proposals in the TPPG has nothing to do with motivation to lower the position of the proposer but is purely based on fairness and altruism; this is because in the TPPG, the display of dominance is not directed at the participants themselves but at another person. Therefore, punishment in the TPPG was not associated with 5-HTTLPR.

This finding that 5-HTTLPR is associated with spiteful rather than altruistic-based punishment may be unsurprising given the function of serotonin in previous research. Serotonin is a neurotransmitter involved in impulsivity and aggression (Montoya et al., 2012). Many studies, including animal studies, have shown that low serotonin levels in the brain are associated with increased aggression (Moeller et al., 1996; Cleare and Bond, 1997; Iofrida et al., 2014; Kästner et al., 2019; da Cunha-Bang and Knudsen, 2021). Unlike altruistic-based punishments, spiteful punishments can be viewed as aggressive behaviors because they are primarily aimed at harming others (Jensen, 2010; Yamagishi et al., 2017). In this light, the association between 5-HTTLPR and salivary punishment is a satisfying result.

Crockett et al. (2010a) argued that participants rejected unfair offers in the UG because lower serotonin levels lead to a loss of emotional control. Indeed, the association between unfair offers and emotional expression in the UG has been frequently reported (e.g., Sanfey et al., 2003; Van’t Wout et al., 2006). However, punishment in the TPPG is reportedly associated with emotional expression (Nelissen and Zeelenberg, 2009; Hartsough et al., 2020); therefore, it is difficult to explain it simply in terms of emotional regulation. One possibility is that unfair offers in the UG and TPPG cause different emotions and that serotonin is only involved in the regulation of the emotions aroused in the UG. Recent studies have shown that unfair offers in the UG cause anger, while unfair offers in the TPPG cause moral outrage (Hartsough et al., 2020). Another study has also shown that the association between injustice harm and intentionality and third-party punishment is mediated by righteous indignation, not anger (Ginther et al., 2021). There is no doubt that both affective and cognitive processing are involved in the behavior toward unfair distribution in the UG and TPPG, but it has also been shown that more affective processing is central in the UG, and conversely, more cognitive processing is central in the TPPG.

At the level of brain functioning, it has been shown that rejection of unfair offers in the UG is related to the activation of emotional regions such as the amygdala and insular cortex (Sanfey et al., 2003; Tabibnia et al., 2008; Gospic et al., 2011), while punishment in the TPPG is the result of complex processes in those regions as well as in regions related to cognitive abilities (temporal-parietal junction, medial prefrontal cortex), the so-called theory of mind, and regions for implementing third-party punishment (dorsolateral prefrontal cortex) (Buckholtz and Marois, 2012). Thus, since punitive behaviors in the UG and TPPG differ in many ways, both in their emotional expression and processing, the association with 5-HTT may be influenced by such differences. Regarding the relationship between 5-HT and emotions, 5-HT has been shown to be associated with anger (Klasen et al., 2019), and some studies have linked polymorphisms in the 5-HTTLPR to activity in the amygdala, a region associated with anger processing (Munafò et al., 2008). In addition, it has been reported that anger expression is also associated with genetic polymorphisms related to tryptophan, the precursor of 5-HT (Alia-Klein et al., 2020). Regarding the relationship between 5-HT and moral outrage, Crockett et al. (2010a) reported a relationship between 5-HT and moral judgment. However, this study did not directly examine the relationship between anger expression and serotonin when making moral judgments. It is still unclear how the anger of third parties relates to serotonin.

Since a previous study reported different rates of 5-HT uptake in SS/SL and LL of 5-HTTLPR (Lesch et al., 1996), the results of the present study may not be interpreted by differences in 5-HT uptake. However, there are clear cultural differences in the proportions of the 5-HTTLPR genotypes, and studies in Asians have reported differences in behavior between SS and SL/LL groups (Gelernter et al., 1997; Chiao and Blizinsky, 2010; Ishii et al., 2014). In the present study, the results in the UG were consistent with the results of studies with non-Asians; hence, it is possible that being homozygous for the L allele is important for non-Asians and being homozygous for the S allele is important for Asians. To support this idea, it would be necessary to examine not only the relationship between behavior and genetic polymorphisms, but also the gene expression levels of Asian participants.

### Limitations

Although we found an association between 5-HTTLPR and the behavioral response to unfair offers in the UG, the mechanism between genes and behavior remains unknown. Studies examining genes and behaviors have attempted to identify the mechanisms between brain volumes and functions by treating them as intermediate phenotypic systems (Tost et al., 2010; Saito et al., 2014; Nishina et al., 2018). A previous study showed that rejection of unfair offers in the UG is related to the activity of the amygdala (Gospic et al., 2011). In addition, a meta-analysis of 14 articles showed a significant association between amygdala activity and 5-HTTLPR (Munafò et al., 2008). Therefore, in future studies, it will be necessary to focus on the activity of the amygdala and investigate the relationship between punishment and serotonin.

This study examined the association between a genetic polymorphism of 5-HTTLPR and behavioral responses to unfair distribution. However, a previous study showed that a single nucleotide polymorphism named rs25531 in the region of 5-HTTLPR has a role in regulating 5-HTTLPR function. Specifically, when the base of rs25531 is G, the function of the L-allele is similar to that of the S-allele (Greenberg et al., 1999; Hu et al., 2005; Armeli et al., 2008). Therefore, in order to rigorously investigate the association between 5-HTTLPR and punishment, rs25531 should be examined in future studies, and its polymorphism should be included in the analysis.

## Conclusion

In the present study, we examined the association between polymorphisms in 5-HTTLPR and behavioral responses to unfair suggestions and found that individuals with the L-allele were more likely to reject unfair proposals in the UG than those with the SS genotype. However, this pattern was not observed in the TPPG. These results indicate that 5-HTT is associated with behavior when unfair intentions are directed at oneself. This finding provides a new perspective on the association between 5-HTT and behavioral responses to unfair distributions. As the present study revealed only the association between genes and behavior, it will be necessary to conduct a comprehensive study that includes the motivation and brain functions behind behavioral responses to unfairness.

## Data Availability Statement

The original contributions presented in the study are included in the article/Supplementary Material, further inquiries can be directed to the corresponding author.

## Ethics Statement

All experimental protocols were approved by the Ethics Committee of Tamagawa University. The protocol for genetic analysis was approved by the Ethics Committee of the Graduate School and Faculty of Medicine, Kyoto University. All participants submitted their consent forms in advance.

## Author Contributions

HiT, MI-M, MS, and HaT designed the research. KN and HaT performed the research. KN, MI-M, and HaT analyzed the data. KN, QS, and HaT wrote the manuscript. All authors contributed to the article and approved the submitted version.

## Conflict of Interest

The authors declare that the research was conducted in the absence of any commercial or financial relationships that could be construed as a potential conflict of interest.

## Publisher’s Note

All claims expressed in this article are solely those of the authors and do not necessarily represent those of their affiliated organizations, or those of the publisher, the editors and the reviewers. Any product that may be evaluated in this article, or claim that may be made by its manufacturer, is not guaranteed or endorsed by the publisher.
